# Hypoglycemia Impairs the Heat Shock Protein Response: A Risk for Heat Shock in Cattle?

**DOI:** 10.3389/fvets.2022.822310

**Published:** 2022-02-10

**Authors:** Samuel A. Atkin, Abu Saleh Md Moin, Stephen L. Atkin, Alexandra E. Butler

**Affiliations:** ^1^School of Veterinary Medicine, University of Liverpool, Liverpool, United Kingdom; ^2^Royal College of Surgeons in Ireland Bahrain, Adliya, Bahrain

**Keywords:** HSP70, HSP27, heat shock protein (HSP), HSP90 (heat shock protein 90), hypoglycemia

## Abstract

**Background:**

Heat stress (HS) in cattle is a major debilitating problem, affecting health and milk yield. Physiologically, HS has been shown to lower blood glucose levels to 2.5 mmol/l (45 mg/dl) and results in upregulation of heat shock proteins (HSPs), eliciting the heat shock response (HSR) of which HSP90, 70 and 27 have been shown to be protective. However, it is unclear if the HSP response is blunted by decreased glucose, thereby preventing adaptive mechanisms. To address this question, this exploratory reverse translational study on the effects of hypoglycemia on the HSP pathway was undertaken.

**Methods:**

A human prospective, study in healthy control individuals (*n* = 23) was undertaken. Subjects underwent hyperinsulinemic-induced hypoglycemia [≤2.0 mmol/L (36 mg/dl)] with blood sampling at baseline, at hypoglycemia and for a 24-h post-hypoglycemia follow-up period. Proteomic analysis of the heat shock-related protein pathway, the pathway associated with HS in cattle, was performed.

**Results:**

In response to hypoglycemia, HS pathway proteins were significantly decreased (*p* < 0.05): HSP70 and HSP27 (at hypoglycemia); DnaJ homolog subfamily B member 1 (DNAJB1), Stress-induced-phosphoprotein 1 (STIP1) and the ubiquitin pathway proteins, Ubiquitin-conjugating enzyme (UBE2L3) and Ubiquitin-conjugating enzyme E2 N (UBE2N) (at 30-min post-hypoglycemia); HSP90 (at 2-h post-hypoglycemia). STIP1, UBE2L3, and UBE2N remained suppressed at 24-h.

**Conclusion:**

Heat stress in cattle reduces blood glucose that, in turn, may blunt the HS pathway protective response, including HSP 90, 70, 27 and the ubiquitin proteins, leading to adverse outcomes. Monitoring of blood glucose in susceptible cattle may allow for earlier intervention and may also identify those animals at greatest risk to ensure that milk yield is not compromised.

## Introduction

Climate change represents a global threat, with (1) increasing temperatures and frequent extreme weather events directly affecting humans. In addition, climate change negatively impacts the animals and crops that are needed to sustain an increasing human population. According to the Intergovernmental Panel on Climate Change (IPCC), the earth warmed by 0.85°C over the period from 1880 to 2012 (2) and, over the next 20 years, the global temperature is expected to reach or exceed 1.5°C of warming (3). Concurrently, the global population is expected to increase by 2 billion in the next 30 years (3) with the need to produce 70% more food by 2050 (4). The future demand for agricultural products highlights the necessity for efficient animal husbandry; however, global warming may have a direct negative impact upon this.

Heat stress in cattle remains a major issue that is consequent upon high ambient temperatures, leading to lower milk production, metabolic diseases such as rumen acidosis, poor milk quality, and lower production performance (5). It has been reported that, in those animals suffering from HS, the heat shock protein (HSP) response, specifically involving increased HSP 90, 70, and 27, acts as a protective mechanism. Further, it has been suggested that HSP70 is a biological marker for quantifying HS in animals (6).

**Figure 1:**
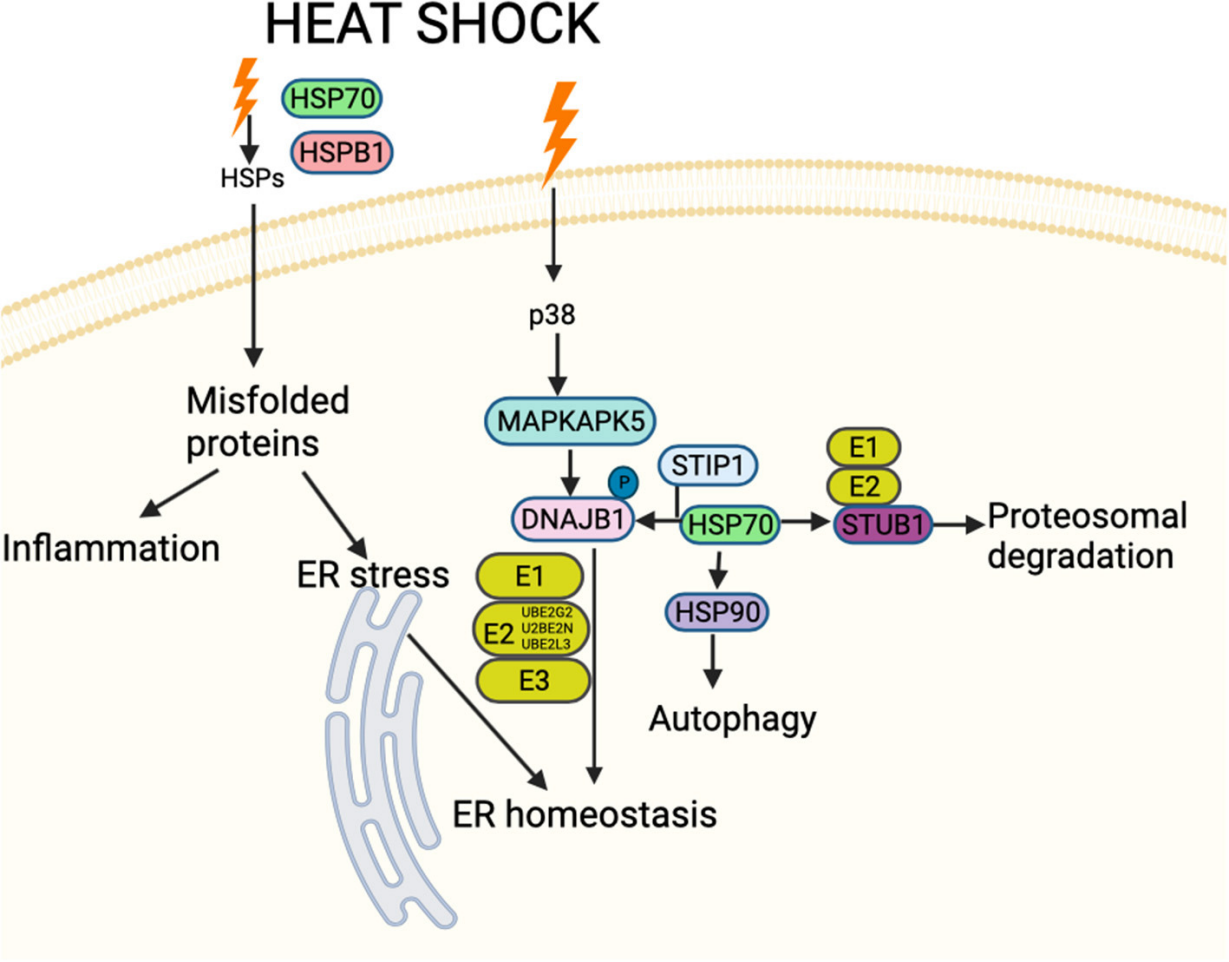
Schematic figure showing an overview of interactions between proteins involved in HSP and associated stress-response pathways likely involved in cattle heat stress. These interactions decide the fate of the downstream signaling pathway. The HSPs and associated proteins interact with the cell surface receptors and/or with each other in response to different stimuli, including accumulated unfolded/misfolded proteins, hormones and cellular/environmental stress (including cattle heat stress) and regulate different molecules affecting a spectrum of biological functions such as apoptosis, autophagy, cell migration and alterations in the immune response. Schematic created using Biorender (https://Biorender.com).

HSPs are categorized by molecular weight (7) and their pathways are complex. Following a heat shock event, DnaJ homolog subfamily B member 1 (DNAJB1: HSP40) may be activated by MAP kinase-activated protein kinase 5 (MAPKAPK5), leading to upregulation of HSP70 and HSP90 that form a complex with Stress-induced-phosphoprotein 1 (STIP1), ultimately leading to activation of the ubiquitin system (Figure 1). The unfolded protein response (UPR) is critically important for the cellular apparatus to effect clearance of short-lived, damaged and misfolded proteins. Degradation of damaged proteins is accomplished through a coordinated series of actions of three enzymes: ubiquitin-activating enzyme (E1), ubiquitin-conjugating enzyme (E2) and ubiquitin-protein ligase (E3) (Figure 1). Following ubiquitination, proteolysis then proceeds through the 26S proteasome (8). HSPs suppress protein aggregation and help with folding and stability of both new and damaged proteins; they chaperone proteins to specific cellular compartments and direct irreversibly damaged proteins for degradation. These processes occur in physiological as well as stressful conditions (9). HSPs bind and control the activity of critical enzymes involved in inflammation, apoptosis, metabolism and cell signaling (10).

Heat stress in cattle is associated with blood glucose values below 2.5 mmol/l (45 mg/dl) (11), but it is unknown what the impact of lower blood glucose levels are on the HS pathway response. Therefore, the proteomic data from a study on hypoglycemia in humans was applied in a reverse translational study to address this question, focusing on the protein pathway encompassing the proteins previously identified in cattle HS (Figure 1).

## Methods

### Study Design

“Healthy control (*n* = 23) Caucasian subjects, aged between 40 and 70 years, were enrolled in a case-controlled study investigating platelet dysfunction to hypoglycemia. All subjects had a body mass index (BMI) between 18 and 49 kg/m^2^, normal renal and hepatic biochemical indices and no prior history of cancer, nor any contraindication to insulin infusion to achieve hypoglycemia (ischemic heart disease, epilepsy, seizure history, drop attacks, history of adrenal insufficiency, and treated hypothyroidism). Blood samples were collected and were measured at the Chemistry Laboratory, Hull Royal Infirmary, UK and measured by an immunometric assay with fluorescence detection on the DPC Immulite 2,000 analyzer using the manufacturer's recommended protocol.

All participants had a medical history, clinical examination (neurological system, cardiovascular, abdominal, musculoskeletal), routine blood tests and an electrocardiogram performed. A continuous insulin infusion was performed to induce hypoglycemia, as previously detailed (12), with blood samples taken at hypoglycemia (time 0), 30-min, 1-h, 2-h and 4-h post-hypoglycemia. After 4-h, participants were provided lunch. Subjects reattended 24-h following the induction of hypoglycemia. Prior to discharge, blood glucose was checked using a glucose analyzer (HemoCue^®^ glucose 201+, Ängelholm, Sweden) to ensure normal levels, together with blood pressure. All participants provided written informed consent. The trial was approved by the North West-Greater Manchester East Research Ethics Committee (REC number: 16/NW/0518), registered at www.clinicaltrials.gov (NCT03102801) and conducted according to the Declaration of Helsinki.”

### Biochemical Markers

Blood samples were separated immediately by centrifugation at 2,000 g for 15 min at 4°C, and the aliquots were stored at −80°C, within 30-min of blood collection, until Somascan batch analysis. Blood samples were collected and were measured at the Chemistry Laboratory, Hull Royal Infirmary, UK and measured by an immunometric assay with fluorescence detection on the DPC Immulite 2,000 analyzer using the manufacturer's recommended protocol.

### Insulin Infusion

**Figure 2:**
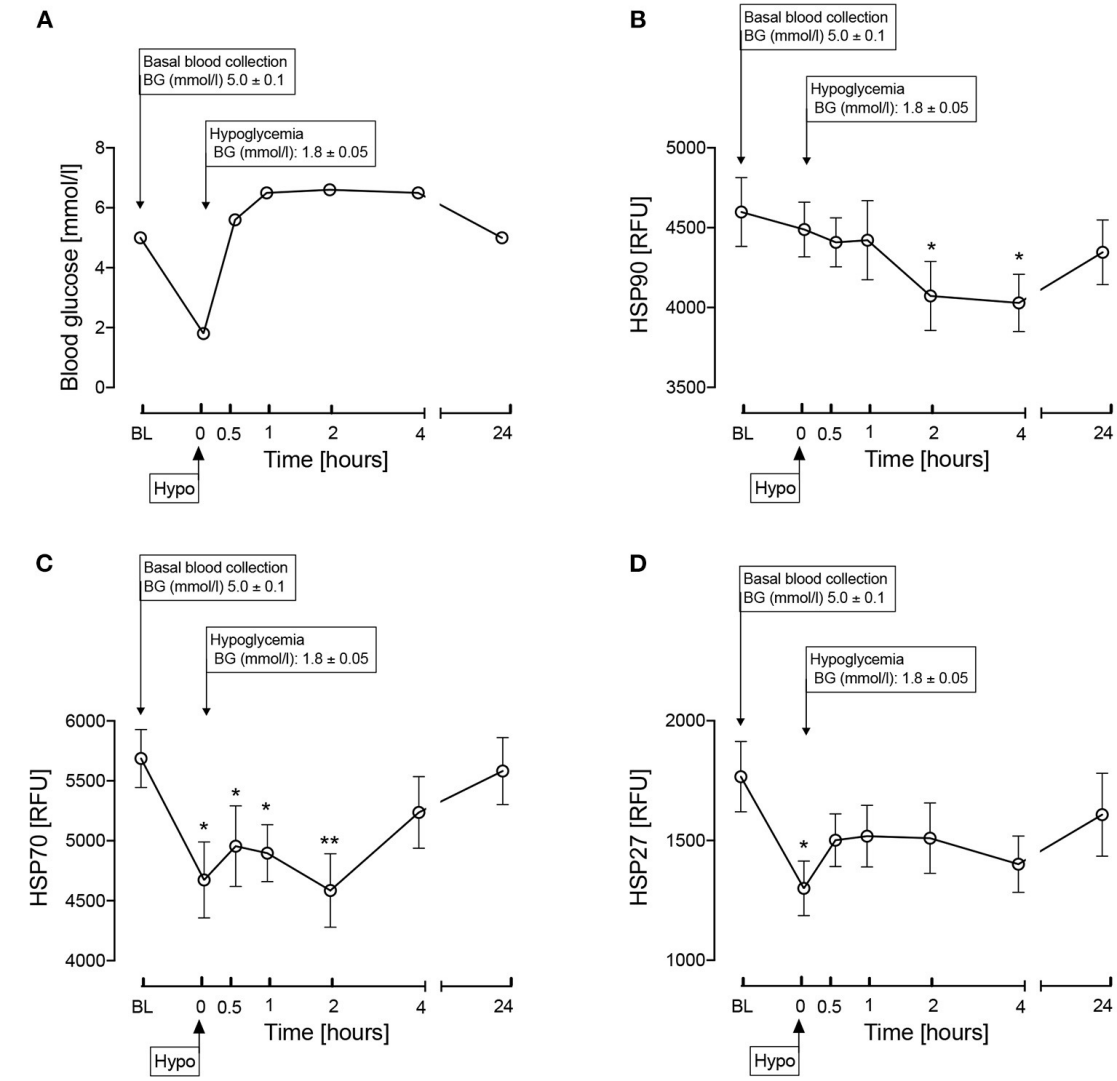
Comparison of blood glucose levels **(A)** and the heat shock proteins, HSP 90 **(B)**, HSP70 **(C)**, HSP27 **(D)** during and after iatrogenic induction of hypoglycemia for normal human subjects. Blood sampling was performed at baseline (BL), at hypoglycemia (0 min) and post-hypoglycemia (30 min, 1-h, 2-h, 4-h, and 24-h). Proteomic (Somalogic) analysis was undertaken for heat shock proteins. Significant changes detailed from baseline *p < 0.05, **p < 0.001.

The insulin infusion was performed as previously detailed (12). “Following an overnight fast, bilateral ante-cubital fossa indwelling cannulas were inserted 30–60 min prior to the commencement of the test (0830h). To induce hypoglycemia, soluble intravenous insulin (Humulin S, Lilly, UK) was given in a pump starting at a dose of 2.5 mU/kg body weight/min with an increment of 2.5 mU/kg body weight/min every 15 min by hypoglycemic clamp (13), until two readings of capillary blood glucose measured by a glucose analyzer (HemoCue^®^ glucose 201+) ≤2.2 mmol/L (<40 mg/dl) or one reading of ≤2.0 mmol/L (36 mg/dl) was achieved (13). The blood sample schedule was timed subsequently in respect to the time point that hypoglycemia occurred. Following the identification of hypoglycemia [as defined by American Diabetes Guidelines (14)], intravenous glucose was given in the form of 150 ml of 10% dextrose and a repeat blood glucose check was performed after 5 min if blood glucose was still <4.0 mmol/L.” Comparison of plasma glucose levels at baseline, at hypoglycemia and post-hypoglycemia timepoints up to 24-h is shown in Figure 2A.

### SOMA-Scan Assay

“To summarize the technology, DNA aptamers named SOMAmers (slow off-rate modified aptamers) were constructed for each protein and quantified using DNA microarray technology. SOMAmers have stable chemical structures and recognize proteins with high binding affinities. Plasma samples were incubated with a mixture of SOMAmers to generate SOMAmer-protein complexes, washed to remove unbound SOMAmers and proteins using bead-based immobilization, following which SOMAmers were then eluted and quantified by hybridizing to a DNA microarray. The SOMAmer mixture quantitatively reflects the original protein concentration.”

“Slow Off-rate Modified Aptamer (SOMA)-scan plasma protein measurement (15) was used to determine a panel of heat shock proteins that were specifically related to HS in cattle, namely heat shock protein-90-alpha/beta (HSP90AA1/HSP90AB1: HSP90), heat shock protein-70 (HSPA1A: HSP70) and heat shock protein-beta-1 (HSPB1; HSP27). The SOMAscan assay used to quantify proteins was performed on an in-house Tecan Freedom EVO liquid handling system (Tecan Group, Maennedorf, Switzerland) utilizing buffers and SOMAmers from the SOMAscan HTS Assay 1.3 K plasma kit (SomaLogic, Boulder, CO) according to manufacturer's instructions and as described previously (16, 17). The assay was performed in 96-well plates containing up to 85 plasma samples, 3 quality control and 5 calibrator plasma samples. Briefly, EDTA plasma samples were diluted into bins of 40, 1, and 0.05% and incubated with streptavidin-coated beads immobilized with dilution-specific SOMAmers *via* a photocleavable linker and biotin. After washing bound proteins were first biotinylated and then released from beads by photocleaving the SOMAmer-bead linker. The released SOMAmer-protein complex was treated with a polyanionic competitor to disrupt unspecific interactions and recaptured on the second set of streptavidin-coated beads. Thorough washing was performed before 5′ Cy3 fluorophore labeled SOMAmers were released under denaturing conditions, hybridized on microarray chips with SOMAmer-complementary sequences, and scanned using the SureScan G2565 Microarray Scanner (Agilent, Santa Clara, CA): each was performed in triplicate.”

### Data Processing and Analysis

“Initial Relative Fluorescent Units (RFUs) were obtained from microarray intensity images using the Agilent Feature Extraction Software (Agilent, Santa Clara, CA). Raw RFUs were normalized and calibrated using the software pipeline provided by SomaLogic. This included (a) microarray hybridization normalization based on spiked-in hybridization controls, (b) plate-specific intensity normalization, (c) median signal normalization, and (d) median calibrator scaling of single RFU intensities according to calibrator reference values. Samples with a high degree of hemolysis (Haptoglobin log RFU < 10) were excluded from the analysis.

Statistical analyses were performed on log_2_ RFU values using R version 3.5.2 (R Foundation for Statistical Computing, Vienna, Austria) including base R package. Data handling and differential protein expression were analyzed using the autonomics and limma (18) packages. For differential protein analysis, we applied limma models containing contrasts between timepoints. Blocking by patient ID was performed to account for random effects. Batch effect correction was performed by adding batch as a covariate to the model. Limma obtained *P*-values were corrected using the Benjamini-Hochberg method (19).”

### Statistical Analysis

There are no studies detailing the changes in HSP response to hypoglycaemia on which to base a power calculation. Sample size for pilot studies has been reviewed by Birkett and Day (20). They concluded that a minimum of 20 degrees-of-freedom was required to estimate effect size and variability. Hence, we needed to analyze samples from a minimum of 20 patients. Data trends were visually evaluated for each parameter and non-parametric tests were applied on data that violated the assumptions of normality when tested using the Kolmogorov-Smirnov Test. Changes from baseline, and from hypoglycemia, to each subsequent timepoint were compared using Student's *t*-test. Statistical analysis was performed using Graphpad Prism (San Diego, CA, USA).

For the proteomic analysis we fitted an intercept-free general linear model as a function of a subgroup (i.e., condition:timepoint), while taking the patient ID as a random effect using the R package limma. Subsequently, we computed the *p*-value for two contrasts: baseline to hypoglycemia for controls, and false discovery rate (FDR) corrected at a value of <0.05 as the cutoff for significance.

## Results

Fasting plasma glucose at baseline was 4.9 ± 0.1 mmol/l. The changes following hypoglycemia in HSPs known to be involved in cattle HS are shown in Figures 2B–D. Following hypoglycemia, HSP90 was significantly decreased (*p* < 0.05) at 2 and 4-h, though had returned to baseline levels by 24-h. At hypoglycemia, HSP70 was significantly decreased (*p* < 0.05) and remained so for 2-h, before then recovering to baseline; HSP27 was significantly decreased at hypoglycemia (*p* < 0.05) with subsequent recovery to baseline values.

**Figure 3:**
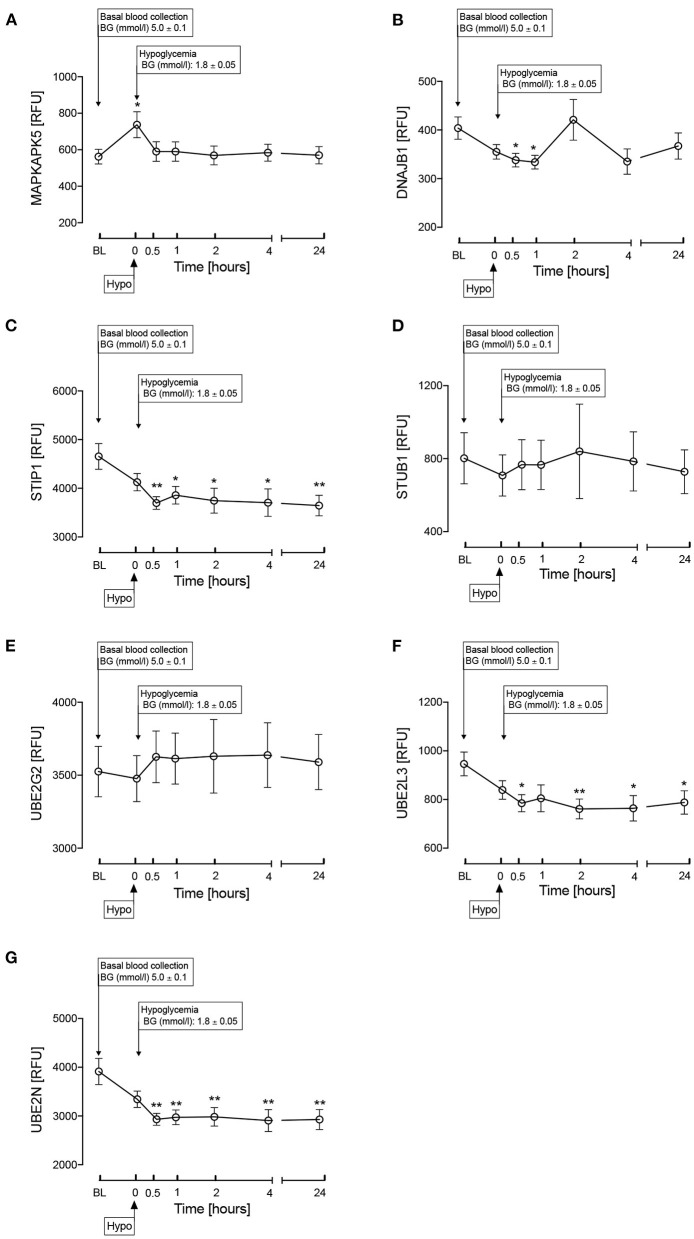
Heat shock protein associated proteins in the HSP pathway as shown in Figure 1. MAPKAPK5 **(A)**, DNAJB1 (HSP40) **(B)**, STIP1 **(C)**, STUB1 **(D)**, UBE2G2 **(E)**, UBE2L3 **(F)**, UBE2N **(G)** during and after iatrogenic induction of hypoglycemia in normal human subjects. Blood sampling was performed at baseline (BL), at hypoglycemia (0 min) and post-hypoglycemia (30 min, 1-h, 2-h, 4-h, and 24-h). Proteomic (Somalogic) analysis was undertaken for heat shock proteins. Significant changes detailed from baseline *p < 0.05, **p < 0.001.

Other proteins involved in the HSP pathway (Figure 1) were then investigated and are shown in Figure 3. MAPKAPK5 increased at hypoglycemia (*p* < 0.05) and normalized thereafter (Figure 3A); DNAJB1 was decreased at 30-min and 1-h post-hypoglycemia (*p* < 0.05), normalizing thereafter (Figure 3B); STIP1 was decreased at 30-min post-hypoglycemia (*p* < 0.05) and remained decreased throughout the 24-h follow up period (Figure 3C); E3 ubiquitin-protein ligase CHIP (STUB1) was unchanged throughout the study time course (Figure 3D), as was the ubiquitin pathway protein, Ubiquitin-conjugating enzyme E2 G2 (UBE2G2) (Figure 3E); the other ubiquitin pathway proteins, UBE2L3 and UBE2N, both decreased at 30-min post-hypoglycemia (*p* < 0.05) and remained so throughout the 24-h follow up period (Figures 3F,G).

## Discussion

Increased HSPs, HSP90, 70, and 27, have been shown to be protective in cattle HS (5); however, in this study, induced hypoglycemia caused a reduction in circulating levels of these three HSPs. HSP27 was decreased significantly only transiently at the point of hypoglycemia and, therefore, is perhaps less impactful on the overall HSP response to hypoglycemia. HSP70 was decreased by hypoglycemia and required a more prolonged recovery time (4-h) to reattain baseline values. HSP 90 showed a slower response to the hypoglycemic insult, its level being decreased at the 2 and 4-h timepoints; given that there were no measurement timepoints in between 4 and 24-h, it is not precisely known when basal levels were reattained.

In animals suffering from HS, HSP90, 70, and 27 are elevated as a protective mechanism and it has been suggested that HSP70 is the ideal biological marker for quantifying HS in animals (6). However, the decrease in blood glucose, as shown here, may reduce or even reverse the HSP protective effect, depending upon the degree of blood glucose lowering. The study reported here confirms such a response but also extends the knowledge of the response that has not been detailed before in the bovine literature with the reduction in ubiquitin pathway proteins, Ubiquitin-conjugating enzyme (UBE2L3) and Ubiquitin-conjugating enzyme E2 N (UBE2N), that remained suppressed at 24-h indicating, that the cellular stress has likely not have resolved.

HSP70 and HSP90 families are involved in protection of the proteome from stress that ensures quality control of protein folding by targeting and direction of misfolded proteins for either re-folding or degradation (21) leading to activation of the ubiquitin system. HSP27 acts as a protein chaperone and an antioxidant, with a role in the inhibition of apoptosis and actin cytoskeletal remodeling (22).

Much of the HSP pathway has not been investigated in cattle heat shock and, therefore, the changes described here in the HSP pathway are speculative; however, the changes relating to HSP27, 70, and 90 reflect those seen in the other proteins. MAPKAPK5 was increased by the lowering of glucose, that was reflected in a decrease in DNAJB1 (HSP 40), STIP1, HSP27, HSP70, HSP90 and in a decrease of the ubiquitin proteins, UBE2L3 and UBE2G2. The changes in the ubiquitin proteins may be of particular importance, as UBE2L3 and UBE2G2 are ubiquitin-conjugating enzyme modifying proteins necessary for misfolded protein degradation; UBE2G2 interacts with the U-box of C terminus of the HSP70 (23). It needs to be ascertained if cattle HS is related to changes in the ubiquitin proteins and whether the severity of the stress is reflected in prolonged changes that were seen here at 24-h.

STUB1 was unchanged with glucose levels that was surprising given that it interacts with HSP90 that was reduced. STUB1 is an E3 ubiquitin ligase that is chaperone-dependent and interacts with HSP70 and HSP90 to mediate the ubiquitination and proteasomal degradation of receptors, such as Toll-like receptor 4 (24), suggesting that this pathway may not be activated (Figure 1).

There are timepoints in the production life cycle of a dairy cow when there is an increased risk for hypoglycemia. At parturition, cows undergo significant metabolic and endocrine changes to meet the protein and energy demands required for milk production, particularly as modern breeding is directed toward maximum milk yield. The increased demand for milk production, together with dry matter intake of dairy cows being at its lowest at parturition, results in the cow mobilizing its own body fat, protein and mineral reserves to support milk production (25). Although cows respond to this imbalance by increasing dry matter intake after parturition, there is a period of weeks where the cow remains in a negative energy balance (25). Given that glucose is the primary monosaccharide converted into lactose, cows in this period of their production cycle are often hypoglycemic (26). The drop in blood glucose around parturition may be worsened by improper ration formulation, inappetence or systemic disease; however, these same factors could affect glycemia in the cow at any point in the production cycle. Cows suffering from incorrect husbandry or systemic disease are already likely to be hypoglycemic and may be prone to HS in higher ambient temperatures. This leaves them at risk for metabolic diseases, such as rumen acidosis, which may be fatal, and decreased milk production or reproductive performance, resulting in their culling.

The use of animal models that are then applied to model human disease are well-established though it is accepted that there will be species-dependent differing responses. The converse, of applying human data to model an animal disease, is less common though the diagnosis and treatment of human disease such as diabetes is applied to dogs (27). In this case, the heat shock response is a phylogenetically ancient response that is recognized to be a universal response in all organisms from insects such as drosophila to complex mammals and humans (28). The heat shock response, designed for protection from cellular stress, follows the same pathways and with the same proteins universally and therefore it would not be unreasonable to extrapolate the more detailed data from a human HSP study to that of an animal HS condition, as here, that would allow the generation of future targeted animal research.

A limitation of this reverse translational study is that the work was performed in humans rather than cows and that the measurement of plasma HSPs may not reflect tissue level expression. Whilst these are novel data, validation is needed through further studies to repeat this work in cattle using a larger sample size specifically looking at the HSPs following graded hypoglycemia from 2.5 mmol/l (45 mg/dl) (11) to define the specific HSP activation cutpoint that may advise cattle husbandry. The use of continuous glucose monitoring in cattle (29) in differing conditions would further define those that may be predisposed to hypoglycemia and the induction of HS.

In conclusion, hypoglycemia reduced HSP90, 70, and 27 and the ubiquitin proteins, that may reflect a reduction in their protective effect in cattle HS, leading to adverse outcomes. Proactive monitoring of blood glucose in susceptible cattle may lead to prevention of cattle HS and may also identify those animals at greatest risk for its development.

## Data Availability Statement

The raw data supporting the conclusions of this article will be made available by the authors, without undue reservation.

## Ethics Statement

The studies involving human participants were reviewed and approved by North West-Greater Manchester East Research Ethics Committee. The patients/participants provided their written informed consent to participate in this study.

## Author Contributions

SAA, AM, and AB analyzed the data and wrote the manuscript. SLA contributed to study design, data interpretation, and the writing of the manuscript. AB is the guarantor of this work. All authors reviewed and approved the final version of the manuscript.

## Conflict of Interest

The authors declare that the research was conducted in the absence of any commercial or financial relationships that could be construed as a potential conflict of interest.

## Publisher's Note

All claims expressed in this article are solely those of the authors and do not necessarily represent those of their affiliated organizations, or those of the publisher, the editors and the reviewers. Any product that may be evaluated in this article, or claim that may be made by its manufacturer, is not guaranteed or endorsed by the publisher.

## Abbreviations

ER: endoplasmic reticulum
SOMA: Slow Off-rate Modified Aptamer
HSPs: heat shock proteins
UPS: ubiquitin proteasome system
UPR: unfolded protein response
HSP70: heat shock protein 70
HSP27: heat shock protein 27
HSP90: heat shock protein 90
DNAJB1: DnaJ homolog subfamily B member 1
MAPKAPK5: MAP kinase-activated protein kinase 5
STIP1: Stress-induced-phosphoprotein 1
E1: Ubiquitin activating enzyme
E2: Ubiquitin conjugating enzyme 2
UBE2G2: Ubiquitin-conjugating enzyme E2 G2
E3: Ubiquitin protein ligase
UBE2L3: Ubiquitin-conjugating enzyme
UBE2N: Ubiquitin-conjugating enzyme E2 N
STUB1: E3 ubiquitin-protein ligase CHIP.
